# Integrating Speciation Analysis and Bioaccessibility to Reassess Cadmium Exposure Risk from Six Mushrooms

**DOI:** 10.3390/toxics14010066

**Published:** 2026-01-10

**Authors:** Peng Liu, Ximei Wang, Wanchao Chen, Yan Yang

**Affiliations:** Institute of Edible Fungi, Shanghai Academy of Agricultural Sciences, National Engineering Research Center of Edible Fungi, Key Laboratory of Edible Fungi Resources and Utilization (South), Ministry of Agriculture and Rural Affairs, Shanghai 201403, China; liupeng@saas.sh.cn (P.L.); ximei_w@163.com (X.W.); chenwanchao@saas.sh.cn (W.C.)

**Keywords:** heavy metal, bioaccessibility, *Agaricus blazei*, sequential extraction

## Abstract

Accurate assessment of dietary exposure to cadmium in mushrooms is crucial for food safety. The inherent limitation lies in relying solely on total cadmium content, failing to reflect its actual bioaccessibility. This study integrated speciation analysis and bioaccessibility to provide a comprehensive risk evaluation. The results showed that cadmium primarily existed in the residual state across *Lentinus edodes*, *Morchella esculenta*, *Cordyceps militaris*, *Lyophyllum decastes*, *Agaricus blazei*, and *Stropharia rugosoannulata*, indicating that a significant portion of the cadmium is tightly bound within insoluble cellular structures, rendering it relatively inert and low mobility. Among them, *A. blazei* exhibited the highest total cadmium (3.84 mg/kg) and contained detectable acid-soluble cadmium. However, the in vitro bioaccessibility of *A. blazei* was low (~6%), and no cadmium was detected in the other five mushrooms after biomimetic digestion, reflecting “high content, low release” characteristics. For *A. blazei*, digestion significantly increased soluble polysaccharides, suggesting that the substantial release of polysaccharides in the gastrointestinal environment not only contributes to their bioactive functions but may also inhibit the dissolution and absorption of cadmium through mechanisms such as adsorption and complexation. Concludingly, this study underscores the necessity of integrating bioaccessibility data for the accurate safety assessment of cadmium in mushrooms.

## 1. Introduction

Edible mushrooms are highly valued in modern healthy diets due to their rich content of proteins, polysaccharides, dietary fibers, and various bioactive compounds. However, during growth, mushroom mycelia possess efficient extracellular chelation and intracellular sequestration mechanisms, often involving metallothioneins and phytochelatins, which enable them to accumulate heavy metal cadmium (Cd) from growth substrates such as soil, compost, or agricultural waste, which poses a significant concern for their dietary safety [1,2,3]. Cadmium is a toxic heavy metal that can accumulate in the human body upon long-term intake, causing damage to the kidneys and bones, and carrying potential carcinogenic risks [4,5,6]. In response, international food safety authorities, such as the Joint FAO/WHO Expert Committee on Food Additives (JECFA) and the European Food Safety Authority (EFSA), have established strict intake guidelines, including a provisional tolerable monthly intake (PTMI) of 25 μg/kg body weight. Many countries have consequently set maximum limits for cadmium in foodstuffs, underscoring the need for accurate exposure assessments. Bioaccessibility describes the fraction that is released from the food matrix into the gastrointestinal juices and becomes soluble and potentially available for intestinal absorption; bioavailability refers to the proportion that is actually absorbed across the intestinal epithelium and enters systemic circulation. For heavy metals like cadmium, the bioaccessible fraction is a critical, though often overlooked, determinant of true dietary exposure, as a large portion of the total metal may remain sequestered in an indigestible form. Most existing studies and regulatory frameworks, however, still rely primarily on total cadmium content, creating a potential gap between estimated and actual risk. While numerous studies have investigated cadmium contamination in edible mushrooms, most have focused primarily on measuring total cadmium content [7,8]. The toxicity and bioaccessibility of cadmium, however, depend not only on its total concentration but also critically on its chemical speciation. Different forms of cadmium exhibit varying solubilities, mobilities, and potential for absorption in the human gastrointestinal tract. Studies have shown significant differences in the leaching rates of cadmium under different pH conditions [9,10]. Therefore, investigating cadmium speciation is essential for a more accurate assessment of its health risks.

*Agaricus blazei* is a medical mushroom that has huge potential commercial value, with various health-promoting functions. Nevertheless, *A. blazei* exhibited a high tolerance to the heavy metal cadmium [11]. It was reported that cadmium accumulation in *A. blazei Murill* has been found to reach 3 to 35 mg/kg dry matter [12]. The cadmium content in the *A. blazei Murill* fruiting body often exceeds the food contaminant limits (2 mg/kg fresh matter) according to National food safety standard—Limits of contaminants in food (GB2762-2022, China, 2022) [13]. However, the fate of cadmium during digestion remains inadequately understood, such as its release from the fungal matrix and potential interaction with other components like proteins and polysaccharides. A key concept in such a risk assessment is “bioaccessibility”, which refers to the fraction of a contaminant that is released from the food matrix into the digestive juices and thus becomes available for potential intestinal absorption. Data on the bioaccessibility of cadmium in mushrooms are still limited.

To address these knowledge gaps, this study aimed to provide a comprehensive, comparative assessment of cadmium exposure risk from edible mushrooms by integrating chemical speciation and bioaccessibility. The system measured and compared the total content and forms of cadmium in the fruiting bodies of *A. blazei* and five other common edible mushrooms, and evaluated the in vitro gastrointestinal bioavailability of cadmium in these species using a biomimetic digestion model. At the same time, the distribution and digestion release of cadmium in different parts of *A. blazei* (cap and stem) were studied, and the dynamic co-release of cadmium with major nutrients (proteins and polysaccharides) during digestion was analyzed to explore potential matrix interactions. This multi-faceted approach was designed to elucidate the key factors controlling cadmium bioavailability in mushrooms and to establish a more scientifically robust basis for their dietary safety evaluation.

## 2. Materials and Methods

### 2.1. Materials

Fruiting bodies of *Lentinus edodes*, *Morchella esculenta*, *Cordyceps militaris*, *Lyophyllum decastes*, and *A. blazei* were obtained from Susen Food Business Department (Guandu District, Kunming, China). Fruiting bodies of *Stropharia rugosoannulata* were procured from Shanghai Gulin Yuan Fungi Professional Cooperative (Shanghai, China). These mushrooms’ fruiting bodies are commonly consumed in culinary practices and are also known in traditional contexts for their health-promoting properties, which are often associated with bioactive constituents such as polysaccharides, proteins, and other compounds. Artificial saliva, gastric juice, and intestinal juice were obtained from Beijing Solarbio Science & Technology Co., Ltd. (Beijing, China). All chemicals used for extractions and analyses, including anhydrous glucose, concentrated sulfuric acid, phenol, concentrated hydrochloric acid, and sodium hydroxide, were of analytical grade and purchased from Sinopharm Chemical Reagent Co., Ltd. (Shanghai, China).

### 2.2. Determination of Total Cadmium Content

The total cadmium content in each mushroom powder was determined in triplicate via graphite furnace atomic absorption spectrometry (GFAAS) following the Determination of cadmium in foods (GB 5009.15-2023, China, 2023) [14]. Briefly, approximately 0.5 g of each powdered sample was accurately weighed and digested overnight with nitric acid in a polytetrafluoroethylene-lined digestion vessel. Subsequently, a 30% hydrogen peroxide solution was added, and digestion was continued at 120~160 °C for 4~6 h. The digested solution was analyzed using a PinAAcle 900Z atomic absorption spectrophotometer (PerkinElmer, Hopkinton, MA, USA) under the following operating conditions: wavelength, 228.8 nm, slit width, 0.2–1.0 nm, lamp current, 2~10 mA, drying at 105 °C for 20 s, ashing at 400~700 °C for 20~40 s, and atomization at 1300~2300 °C for 3~5 s. Quality assurance was performed using certified reference materials and reagent blanks throughout the analytical procedure.

### 2.3. Sequential Extraction Procedure for Cadmium Speciation

A sequential extraction procedure was adopted to determine the chemical forms of cadmium in the mushroom powders. In brief, 10.0 g of each powdered sample was sequentially extracted as follows. (1) Water-soluble fraction: The sample was extracted with 200 mL of deionized water under boiling for 2 h, followed by centrifugation (8000× *g*, 10 min). The residue was re-extracted with 100 mL of deionized water under the same conditions. The combined supernatants were collected as the water-extractable cadmium. (2) Acid-soluble fraction: The residue from step (1) was extracted with 200 mL of 0.1 mol/L HCl at 60 °C for 2 h with shaking, followed by centrifugation (8000× *g*, 10 min). The supernatant represented the acid-extractable cadmium. (3) Alkali-soluble fraction: The residue from step (2) was extracted with 200 mL of 0.1 mol/L NaOH at 60 °C for 2 h with shaking, followed by centrifugation (8000× *g*, 10 min). The supernatant corresponded to the alkali-extractable cadmium. (4) Residual fraction: The final solid residue was digested, and its cadmium content was determined as the residual (insoluble) cadmium. The cadmium concentration in all extracts and the final residue was measured by GFAAS as described in the previous section.

### 2.4. In Vitro Biomimetic Digestion

An in vitro digestion model simulating the human gastrointestinal tract (oral, gastric, and intestinal phases) was employed to assess cadmium bioaccessibility. Briefly, 0.01 g of mushroom powder was mixed with 10 mL of artificial saliva and incubated at 37 °C for 5 min under constant agitation to simulate the oral phase. The resulting bolus was then transferred to 150 mL of artificial gastric juice and incubated at 37 °C for 2 h with agitation (gastric phase). Subsequently, 150 mL of artificial intestinal juice was added, and the mixture was further incubated at 37 °C for 4 h with agitation (intestinal phase). At the end of each digestive phase (oral, gastric, and intestinal), aliquots of the digest were collected, immediately cooled on ice, and centrifuged (10,000× *g*, 15 min, 4 °C). The supernatants were collected and stored for subsequent analysis of cadmium, protein, and polysaccharide content. The bioaccessibility of cadmium was calculated using the following formula: (cadmium content in intestinal phase supernatant/Total cadmium content in undigested sample) × 100%.

### 2.5. Analysis of Protein and Polysaccharide Content

Protein content was determined using the Bradford method [15]. Polysaccharide content was determined using the phenol-sulfuric acid method [16], with D-glucose as the standard. These analyses were performed on both the undigested mushroom powders and the supernatants obtained after the intestinal phase of biomimetic digestion.

### 2.6. Statistical Analysis

All experiments were conducted with at least three independent replicates. Data are presented as mean ± standard deviation (SD). Statistical analysis was performed using GraphPad Prism software (version 10). Differences between the two groups were evaluated using Student’s independent samples *t*-test. For comparisons among three or more groups, one-way analysis of variance (ANOVA) was performed, followed by Tukey’s post hoc test for multiple comparisons. The correlation analysis of cadmium, polysaccharides, and protein content was conducted using Spearman analysis. A *p* < 0.05 was considered statistically significant.

## 3. Results

### 3.1. Cadmium Content Analysis

**Figure 1:**
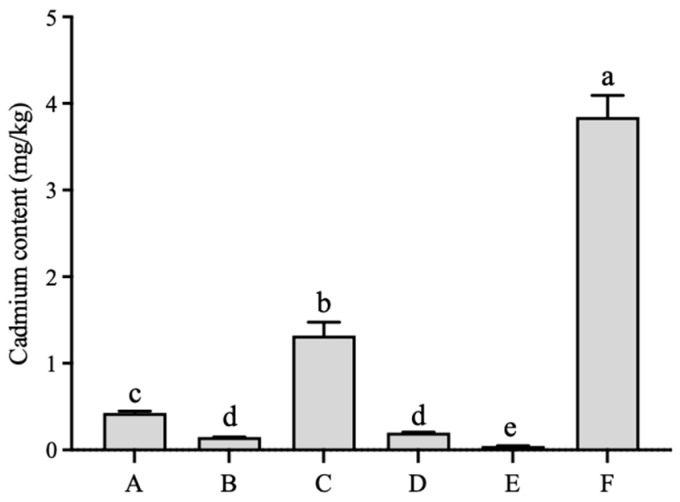
Analysis of cadmium content in different edible mushroom fruiting bodies. A: *L. edodes*; B: *S. rugosoannulata*; C: *M. esculenta*; D: *C. militaris*; E: *L. decastes*; F: *A. blazei*. Different lowercase letters represent significant differences (*p* < 0.05).

The comprehensive analysis of six edible mushroom species revealed significant differences in their cadmium burden. The total cadmium content in the fruiting bodies followed the order *A. blazei* > *M. esculenta* > *L. edodes* > *C. militaris* > *S. rugosoannulata* > *L. decastes* (Figure 1). Notably, *A. blazei* exhibited the highest cadmium concentration (3.84 mg/kg), which was significantly greater than that found in *M. esculenta* (1.31 mg/kg). The cadmium levels in the other four species were all below 1.0 mg/kg. The pronounced cadmium accumulation in *A. blazei* aligns with existing literature highlighting its strong propensity for heavy metal uptake, likely influenced by its commonly used cultivation substrates such as fermented straw, livestock manure, and cottonseed hulls, which may themselves contain cadmium [11,17]. While the cadmium content in five of the six mushrooms was below the national safety limit, the elevated level in *A. blazei* underscores the necessity for continuous monitoring and substrate management for this species.

### 3.2. Analysis of Cadmium Speciation in Fruiting Bodies

Beyond total content, the chemical speciation of cadmium, which dictates its mobility and potential bioaccessibility, was investigated through sequential extraction. A dominant finding across all six species was that the majority of cadmium existed in the residual fraction (Table 1). This indicates that a significant portion of the cadmium is tightly bound within insoluble cellular structures or complexes, rendering it relatively inert and less likely to be released under mild conditions. However, crucial differences emerged in the more labile fractions. While most species showed negligible levels of exchangeable cadmium, *A. blazei* was unique in containing detectable amounts of cadmium in all three extractable forms: acid-soluble (0.224 mg/kg), water-soluble (0.036 mg/kg), and alkali-soluble (0.021 mg/kg). The presence of acid-soluble cadmium is particularly relevant for dietary risk assessment, as this fraction is susceptible to dissolution in the acidic environment of the human stomach.

### 3.3. Biological Accessibility Analysis of Cadmium in Fruiting Bodies

Biological accessibility of cadmium was directly tested using an in vitro biomimetic gastrointestinal model. The results were striking after the complete oral-gastric-intestinal digestion process; cadmium was detected only in the artificial intestinal fluid of *A. blazei*, at a concentration of 0.22 mg/kg, corresponding to a bioaccessibility of 5.73% (Table 2). This low release rate, despite the relatively high total cadmium, reinforces the importance of speciation. It suggests that for most mushrooms, including those with lower total cadmium, the metal remains largely sequestered in the residual matrix during digestion and is likely excreted with the fecal matter, posing minimal absorption risk. The digestion process also profoundly affected the release of major nutritional components. A consistent and remarkable finding across all six mushroom species was a significant increase in polysaccharide content in the intestinal digest compared to the undigested powder (Table 2). For instance, polysaccharides from *L. edodes* increased by over 6-fold. This is likely due to the breakdown of the robust fungal cell wall during gastric and intestinal digestion, liberating encapsulated polysaccharides into the soluble fraction [18,19]. This enhanced release of bioactive polysaccharides is nutritionally favorable. Moreover, numerous studies have also shown that polysaccharides have a good protective effect against cadmium-induced toxicity [20,21]. Importantly, the fact that the released cadmium did not correlate with the massive increase in soluble polysaccharides suggested that cadmium was not primarily associated with these polysaccharide fractions in a bioaccessible form. This dissociation implies that the beneficial health effects of mushroom polysaccharides may be leveraged with a reduced concomitant risk of cadmium co-absorption.

### 3.4. Biological Accessibility Analysis of Cadmium in Different Parts of A. blazei

**Figure 2:**
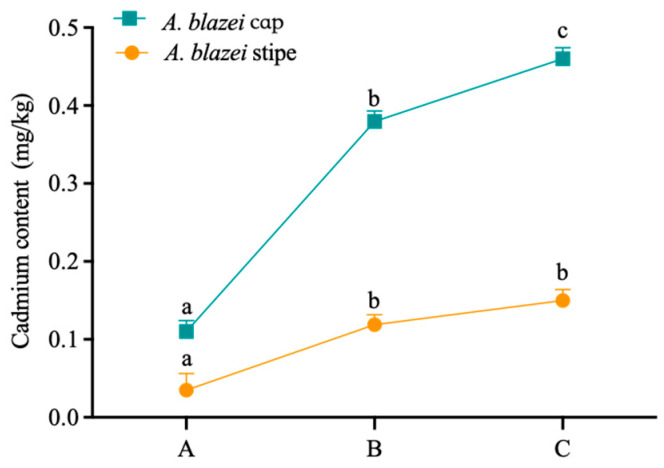
Changes in cadmium content during biomimetic digestion of *A. blazei* stipe and cap. A: artificial saliva juice; B: artificial gastric juice; C: artificial intestinal juice. Different lowercase letters represent significant differences in cadmium content in different biomimetic digestive juices within the same sample (*p* < 0.05).

A deeper investigation into *A. blazei* revealed a clear compartmentalization of cadmium, with the cap (7.65 mg/kg) accumulating nearly three times more cadmium than the stipe (2.58 mg/kg). This distribution pattern was consistent across all chemical fractions, with the cap showing significantly higher concentrations in each. Interestingly, the speciation pattern (predominantly residual, followed by acid-soluble) was similar in both parts, suggesting a consistent binding mechanism throughout the fruiting body, albeit at different intensities (Table 3). Changes in cadmium content during biomimetic digestion of A. blazei stipe and cap showed that the cadmium content in the stem and cap of *A. blazei* gradually increased in biomimetic oral and gastrointestinal digestive juices. Compared to the cadmium content in artificial saliva of fungal stems and caps, the cadmium content in artificial gastric digestive juices significantly increased by 2.4 times and 2.45 times, respectively. The cadmium content in the artificial intestinal digestion solution was 0.15 and 0.46 mg/kg, respectively, with bioaccessibility of 5.81% and 6.01% (Figure 2).

The water extracted components of the stipe and cap of *A. blazei* contained the highest protein and polysaccharide content. In addition, proteins were mainly distributed in the alkaline extraction components (Table 3). This is completely in line with existing knowledge that proteins and polysaccharides are water-soluble components, and their highest proportion in the water extraction component is inevitable [22,23,24]. In both non-biomimetic digestion and biomimetic digestion artificial intestinal fluid, the protein content of the cap was significantly higher than that of the stipe, while its polysaccharide content was significantly lower than that of the stipe. Compared to before biomimetic digestion, the protein content in the artificial intestinal fluid of the stipe and cap decreased by 18.64% and 47.16%, respectively, after biomimetic digestion, while the polysaccharide content increased by 68.57% and 254.66% respectively (Table 4). It can be attributed to the combined effects of proteolytic degradation and possible re-complexation or precipitation during the digestion process. A significant portion of the initial protein may have been hydrolyzed beyond the detection range of the assay or transformed into insoluble aggregates. The acidic gastric phase could induce protein denaturation and aggregation, which may not fully re-solubilize in the intestinal phase, leading to a lower measurable soluble protein fraction post-digestion [25]. This indicates that digestion not only releases nutrients but can also alter their solubility and detectability, impacting the apparent bioaccessibility of proteins.

### 3.5. Correlation Analysis of Cadmium, Protein, and Polysaccharide Content in A. blazei

**Figure 3:**
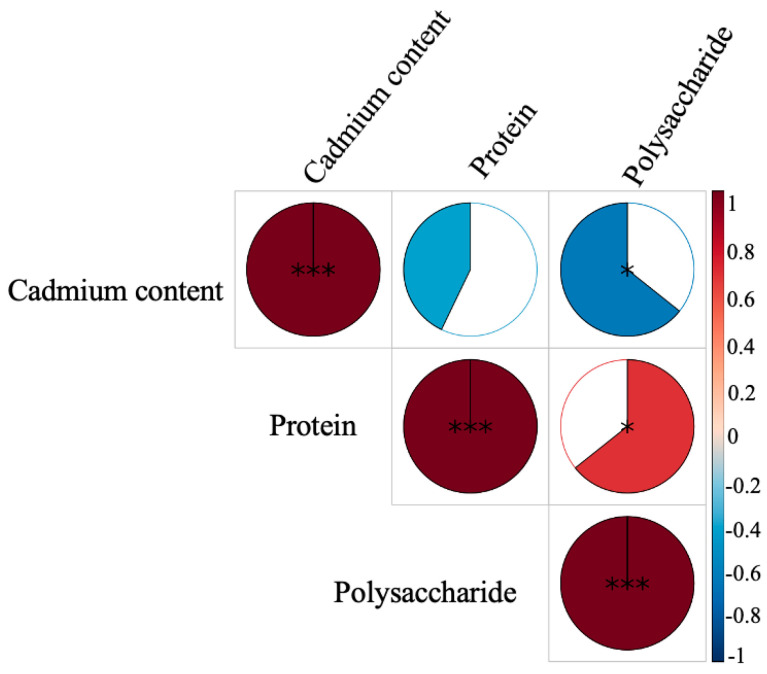
Correlation analysis of cadmium, protein, and polysaccharide content in *A. blazei*. This represents a statistically significant correlation between the two indicators (*: *p* < 0.05, ***: *p* < 0.001).

Correlation analysis showed that a concurrent significant increase in polysaccharide content and a low release rate of cadmium in *A. blazei* were observed, demonstrating a negative correlation between these two factors (Figure 3). This suggests that the substantial release of edible mushroom polysaccharides in the gastrointestinal environment not only contributes to their bioactive functions but may also inhibit the dissolution and absorption of cadmium through mechanisms such as adsorption and complexation. Thereby, while exerting health-promoting effects, these polysaccharides may play the role of a “natural antagonist” in reducing the bioaccessibility and toxicity of heavy metals. Moreover, numerous reports have shown that *A. blazei* polysaccharides could improve cadmium-induced tissue damage through enhancing antioxidant activity and alleviating inflammatory response [26,27]. Our study provides another perspective on the protective mechanism of polysaccharides from *A. blazei* against heavy metal cadmium toxicity, and lays the foundation for the synergistic development of safety and functionality in high-value-added edible mushroom products. It should be emphasized that the mechanism through which polysaccharides may reduce cadmium bioaccessibility was based primarily on the observed inverse correlation between polysaccharide release and cadmium solubilization. This association, while compelling and supported by existing literature on the metal-binding capacity of polysaccharides, remains indirect and hypothetical within the context of our experimental setup. The current study does not provide direct evidence for the formation of cadmium–polysaccharide complexes in the digestive milieu. To unequivocally establish this interaction, future research should employ direct binding assays, spectroscopic techniques (such as FT-IR or X-ray absorption spectroscopy), and fractionation studies to isolate and characterize polysaccharide-bound cadmium. Nevertheless, the consistent negative correlation observed here provides a strong rationale for these more targeted investigations and underscores the potential for mushroom polysaccharides to act as natural mitigants of heavy metal exposure.

## 4. Discussion

The findings of this study collectively underscore a critical paradigm in the dietary risk assessment of heavy metals in edible mushrooms: total concentration is an insufficient predictor of exposure, and integration of metal speciation and bioaccessibility is essential for a realistic evaluation. Our data reveal a consistent pattern across the six cultivated species investigated, wherein the majority of cadmium was found in the residual, biologically inert fraction. This is a reassuring indicator that a significant portion of the accumulated metal is strongly sequestered within insoluble cellular structures, likely complexed with cell wall components like chitin or associated with intracellular granules, thereby limiting its mobility and potential for release during digestion. However, the unique profile of *A. blazei*, which contained measurable acid-soluble cadmium in addition to the dominant residual pool, highlights important interspecies variability. This acid-soluble fraction, though small relative to the total pool, is of particular toxicological relevance as it represents the cadmium pool most susceptible to solubilization in the acidic gastric environment, a process confirmed by the marked increase in cadmium concentration observed in the gastric phase of our in vitro digestion. It was reported that cadmium might be remobilized after the immobilization process through the acid-soluble and complexation effects, which may be the key factor causing cadmium exudation from *A. blazei* in acid solution [28]. The high concentration of H^+^ ions can displace Cd^2+^ ions from adsorption sites on biological matrices like cell walls and proteins [29]. Furthermore, the acidic environment may promote the partial hydrolysis or protonation of structural polysaccharides (e.g., chitin and glucans) in fungal cell walls, increasing porosity and facilitating the release of entrapped metals [30,31]. The subsequent enzymatic action of pepsin, degrading proteins that may bind cadmium (e.g., metallothioneins), could contribute to further mobilization in the stomach [32]. The subsequent low recovery in the intestinal phase, translating to a bioaccessibility of only ~6%, strongly indicates that much of this mobilized cadmium is reabsorbed, reprecipitated, or complexed by other digesta components before it can enter the soluble pool available for absorption.

The central finding of “high content, low release” for *A. blazei* carries significant implications for food safety regulation. When contextualized within an estimated dietary intake scenario, the discrepancy between risk assessments based on total versus bioaccessible cadmium becomes stark. To contextualize our bioaccessibility findings in terms of human health risk, a brief dietary exposure assessment was performed using *A. blazei* as a case study. Assuming an adult (60 kg body weight) consumes 20 g (dry weight) of *A. blazei* daily, the weekly intake of total cadmium would be approximately 5.38 μg/kg bw/week. This value exceeds the PTWI of 2.5 μg/kg bw/week established by JECFA for cadmium. However, when bioaccessible cadmium (5.73% of total cadmium, i.e., 0.22 mg/kg) is used in the same exposure scenario, the estimated weekly intake drops to approximately 0.31 μg/kg bw/week, which is only about 12% of the PTWI. This stark contrast underscores that reliance on total cadmium content would lead to a substantial overestimation of exposure risk, whereas the bioaccessibility-adjusted estimate suggests that consumption of *A. blazei* poses a low risk under the assumed intake conditions. These calculations reinforce the necessity of integrating bioaccessibility data into food safety evaluations, as they provide a more realistic basis for risk assessment and regulatory decision-making regarding heavy metals in edible mushrooms.

A compelling and nutritionally synergistic observation from this study is the substantial increase in soluble polysaccharides during in vitro digestion across all species, concurrent with the minimal release of cadmium. The strong negative correlation observed in *A. blazei* between polysaccharide concentration and cadmium bioaccessibility suggests a potential interaction. It is plausible that the liberated polysaccharide chains, with their abundant hydroxyl, carboxyl, and other functional groups, act as bio-polymers capable of binding cadmium ions through adsorption or complexation, thereby reducing their free concentration and potential for absorption. This hypothesis is supported by a body of literature demonstrating the metal-chelating capacities of various polysaccharides and their protective effects against cadmium toxicity in vivo [20,21]. However, it is crucial to state that within the framework of the present study, this mechanism remains hypothetical and is supported primarily by correlative evidence.

The interpretation of these findings must be tempered by a clear acknowledgment of methodological limitations. The in vitro biomimetic digestion model, while a valuable, reproducible, and ethically favorable tool for screening and comparative assessment, simplifies the complexity of human gastrointestinal physiology. It does not account for the role of the gut microbiota, which can further modify metal speciation and food matrix components, nor for interindividual variability in factors such as pH, transit time, and enzymatic activity. Most importantly, the measured bioaccessibility is not equivalent to bioavailability, which is the fraction that ultimately crosses the intestinal epithelium and enters systemic circulation. Thus, our data represent a conservative estimate of the maximum potentially available cadmium under standardized conditions, and the true systemic uptake in humans may be even lower. Furthermore, the broader generalization of our conclusions requires caution. The low bioaccessibility documented here is characteristic of the specific mushroom species grown under controlled cultivation conditions with the substrates described. Cadmium accumulation and its subsequent behavior are highly species-specific and profoundly influenced by substrate composition and environmental factors. Wild mushrooms or those cultivated on intentionally or heavily contaminated substrates may exhibit dramatically different speciation profiles and bioaccessibility. Therefore, while the integrative assessment approach we advocate is universally applicable, the specific quantitative conclusions regarding risk are valid primarily for the species and cultivation systems studied.

## 5. Conclusions

In conclusion, this study demonstrates that total cadmium content alone is a poor predictor of dietary exposure risk from edible mushrooms. The key determinants are the chemical speciation of cadmium and its bioaccessibility upon digestion. While *A. blazei* accumulated the highest total cadmium content, its low bioaccessibility (~6%) indicates that only a small fraction is released for potential absorption. The dominant residual speciation in mushrooms and the dissociation of released cadmium from beneficial polysaccharides are reassuring findings for consumer safety. These results advocate for the integration of bioaccessibility studies into food safety risk assessments for heavy metals in fungi, providing a more nuanced and accurate understanding of true consumer exposure. Future research should focus on the molecular mechanisms of cadmium binding in fungal tissues and explore agricultural strategies to minimize the uptake of bioaccessible metal forms during cultivation.

## Figures and Tables

**Table 1:** Speciation of heavy metal cadmium in edible mushroom fruiting bodies (mg/kg).

Mushroom Fruiting Bodies	Water Extraction State	Acid Extraction State	Alkali Extraction State	Residue
*L. edodes*	N	0.023 ± 0.001 ^c^	N	0.238 ± 0.002 ^Ac^
*S. rugosoannulata*	N	N	N	0.008 ± 0.001 ^e^
*M. esculenta*	0.017 ± 0.001 ^C^	0.081 ± 0.001 ^Bb^	N	0.830 ± 0.002 ^Ab^
*C. militaris*	N	N	N	0.050 ± 0.001 ^d^
*L. decastes*	N	N	N	N
*A. blazei*	0.036 ± 0.001 ^Ca^	0.224 ± 0.001 ^Ba^	0.021 ± 0.001 ^D^	1.710 ± 0.003 ^Aa^

Notes: Different lowercase letters in the same column represent statistically significant differences (*p* < 0.05). Different capital letters in the same industry represent statistically significant differences (*p* < 0.05). N represents cadmium content < 0.004 mg/kg in mushroom fruiting bodies.

**Table 2:** Changes in cadmium, protein, and polysaccharide content after biomimetic digestion of different edible mushroom fruiting bodies.

Mushroom Fruiting Bodies	Biological Accessibility of Cadmium (%)	Protein (%)		Polysaccharide (%)	
		Before Biomimetic Digestion	After Biomimetic Digestion	Before Biomimetic Digestion	After Biomimetic Digestion
*L. edodes*	N	22.34 ± 0.52 ^c^	24.24 ± 0.48 ^bc^	6.83 ± 0.19 ^c^	49.53 ± 1.75 ^c^*
*S. rugosoannulata*	N	26.86 ± 1.14 ^b^	27.33 ± 0.64 ^b^	17.32 ± 0.85 ^a^	60.02 ± 1.87 ^a^*
*M. esculenta*	N	32.13 ± 0.96 ^a^	37.93 ± 1.43 ^a^*	11.94 ± 0.74 ^b^	55.92 ± 2.04 ^ab^*
*C. militaris*	N	27.77 ± 0.68 ^b^	22.43 ± 0.84 ^c^*	18.91 ± 0.98 ^a^	51.25 ± 1.56 ^bc^*
*L. decastes*	N	21.54 ± 1.11 ^d^	19.09 ± 1.05 ^cd^	19.09 ± 1.29 ^a^	55.21 ± 1.37 ^ab^*
*A. blazei*	5.73 ± 0.04	28.32 ± 0.69 ^b^	26.36 ± 1.18 ^b^	8.47 ± 0.78 ^b^	42.50 ± 1.29 ^d^*

Notes: Different lowercase letters in the same column represent statistically significant differences (*p* < 0.05). * There is a significant difference in the substance content after biomimetic digestion compared to before biomimetic digestion within the same measurement index (*p* < 0.05). N represents cadmium content < 0.004 mg/kg in edible mushroom fruiting bodies.

**Table 3:** Analysis of the speciation of cadmium in the stipe and cap of *A. blazei*.

*A. blazei*		Water Extraction State	Acid Extraction State	Alkali Extraction State	Residue
Cadmium (mg/kg)	Stipe	0.010 ± 0.005 ^d^	0.144 ± 0.010 ^b^	0.029 ± 0.005 ^c^	1.738 ± 0.002 ^a^
	Cap	0.132 ± 0.002 ^d^*	0.542 ± 0.010 ^b^*	0.275 ± 0.007 ^c^*	5.890 ± 0.130 ^a^*
Protein (%)	Stipe	13.27 ± 0.73 ^a^	0.56 ± 0.03 ^c^	5.54 ± 0.28 ^b^	-
	Cap	10.28 ± 0.56 ^a^*	1.50 ± 0.03 ^c^*	7.66 ± 0.34 ^b^	-
Polysaccharide (%)	Stipe	21.63 ± 0.09 ^a^	2.54 ± 0.05 ^c^	3.67 ± 0.08 ^b^	-
	Cap	7.59 ± 0.52 ^a^*	2.04 ± 0.04 ^b^	1.85 ± 0.01 ^b^*	-

Note: Different lowercase letters in the same industry represent statistically significant differences (*p* < 0.05); * There is a significant difference in cadmium content between the cap and stem of the same component (*p* < 0.05).

**Table 4:** Analysis of protein and polysaccharide content in the stipe and cap of *A. blazei* after biomimetic digestion.

*A. blazei*	Protein (%)		Polysaccharide (%)	
	Before Biomimetic Digestion	After Biomimetic Digestion	Before Biomimetic Digestion	After Biomimetic Digestion
Stipe	18.24 ± 0.52	14.84 ± 0.48 *	23.42 ± 0.19	39.48 ± 1.75 *
Cap	38.93 ± 0.69 ^#^	20.57 ± 1.18 *^#^	6.44 ± 0.78 ^#^	22.84 ± 1.29 *^#^

Notes: * There is a significant difference in the substance content after biomimetic digestion compared to before biomimetic digestion for the same measurement index (*p* < 0.05); ^#^: the protein (polysaccharide) content in the representative cap showed a statistically significant difference compared to the stipe (*p* < 0.05).

## Data Availability

The original contributions presented in this study are included in the article. Further inquiries can be directed to the corresponding author.
